# A Transcriptional Analysis Showing the Effects of GH12 Combined with Fluoride for Suppressing the Acidogenicity of *Streptococcus mutans* Biofilms

**DOI:** 10.3390/microorganisms11071796

**Published:** 2023-07-13

**Authors:** Yuhao Zeng, Yu Chen, Chengchen Duan, Xuelian Jiang, Yufei Wang, Linglin Zhang

**Affiliations:** 1 State Key Laboratory of Oral Diseases, National Clinical Research Center for Oral Diseases, West China Hospital of Stomatology, Sichuan University, Chengdu 610017, China; 2 Department of Cariology and Endodontics, West China Hospital of Stomatology, Sichuan University, Chengdu 610017, China

**Keywords:** antimicrobial peptide, phosphotransferase, *Streptococcus mutans*, lactic acid, transcriptome

## Abstract

The acidogenicity of *Streptococcus mutans* is important for caries development. The antimicrobial peptide GH12 can affect the integrity of cellular membranes and the virulence factors of *S. mutans*. Combining GH12 and NaF (GF) efficiently controlled the development of caries, but its mechanisms remained unrevealed. This research intended to verify the effects of GF on the acidogenicity of *S. mutans* biofilms and to reveal the mechanisms. Lactic acid production assays and pH monitoring assays were conducted to investigate the regulatory effects of the GF treatment on the acidogenicity of *S. mutans* biofilms. RNA sequencing and bioinformatics analyses were conducted to screen the transcriptional profile affected by the GF treatment. The results demonstrated the GF group had significantly less lactic acid and maintained the broth’s pH values above 5.0 for longer times. Thereafter, GO/KEGG enrichment analyses and RT-qPCR validation revealed that the GF treatment mainly restrained the expression of genes related to the carbohydrates’ internalization and metabolism. Compared with NaF, the GF treatment further downregulated the carbohydrates transportation genes. Moreover, compared with GH12, the GF treatment affected the membrane’s integrity more significantly. Generally, GF treatment could arrest the acidogenicity of *S. mutans* biofilms, mainly through suppressing carbohydrates transportation and inhibiting overall metabolism.

## 1. Introduction

Dental caries is one of the most common diseases worldwide. As a chronic infectious disease, it imposes a large economic burden on public healthcare [1]. Untreated caries can cause complicated issues that are costly and time-consuming to solve, such as long-term chronic pain, further infection, tooth loss and systemic health risk, and eventually impair the quality of life [2]. This disease is considered to be the undesirable consequence of the interaction between acidogenic bacteria and improper dietary habits over time [3]. Many factors are involved in the onset and development of dental caries, including fluoride exposure, salivary quality, salivary flow, oral hygiene habits and cariogenic bacteria. A variety of caries-related pathogens have been widely studied and are believed to play vital roles in the onset and development of caries. Among all the species found in dental plaque, *Streptococcus mutans* (*S. mutans*) shows superior abilities in acid production, acid resistance and the synthesis of exopolysaccharides (EPS), which determine its irrefutable role in the development of caries [4]. One of the most direct cariogenic factors of *S. mutans* is its acidogenicity [5]. The fluctuation of pH within the plaque and the mineral homeostasis of hard dental tissues are maintained well under healthy circumstances. However, this homeostasis can be disturbed by bad dietary habits. When introduced to extra carbohydrates, the composition of dental plaque can be altered into a pathogenic form. The acidogenic and aciduric bacteria gain a competitive advantage over other species through a massive intake of carbohydrates for producing acid, mainly through specific phosphoenolpyruvate-dependent phosphotransferase (PEP-PTS) pathways [6]. As a result, the overall metabolic direction of dental plaque tilts towards acid production, resulting in a low pH value that causes a net mineral loss [7]. Thus, arresting the acidogenicity of *S. mutans* is of vital importance in halting the development of caries.

One of the widest-applied caries prevention strategies is fluoride. Water fluoridation was once the primary source of fluoride intake decades ago, which made a great contribution to reducing the incidence of caries [8]. Nowadays, various fluoride applications have emerged according to the needs of different situations, such as fluoride mouth rinses for daily use, fluoride gels for professional local application and fluoride supplements for systemic application [9]. The anticaries mechanisms of fluoride can be divided into mineral and intracellular pathways [10]. Briefly, fluoride replaces the hydroxyl group in hydroxyapatite to form fluorapatite, which has better acid resistance and can thus inhibit the process of demineralization. It can also remain in the plaque’s micro-environment in the form of calcium fluoride, which increases the level of calcium retention and promotes the process of remineralization. As well as its function in mineral balance, fluoride also exerts an intracellular effect on the activities of bacteria. Usually, fluoride enters the cells through diffusion in the form of HF, resulting in the accumulation of fluoride and the acidification of cytoplasm, which disturbs glycolysis by inhibiting enolase. The inactivation of enolase thus leads to reduced production of phosphoenolpyruvate (PEP) and therefore suppresses the function of PEP-PTS, which is the main path of glucose internalization. Moreover, the activity of F-ATPase can be inhibited by F^−^ under the existence of Al^3+^, which further limits proton efflux. However, those functions are observed when intracellular fluoride concentration is at least 200 ppm, which is unachievable under the currently applied dosages, especially when bacteria have formed dental plaque to gain extra resistance to environmental factors [11]. It is worth noting that although some scientists suggest increasing the dose of fluoride applied, the anticaries effects seem to be far less than the risks of inducing side effects or toxic effects [12]. Thus, although it has been widely accepted as a mainstay of caries management, fluoride seems to have reached a limit in the prevention of caries.

Antimicrobial peptides (AMPs) are a series of innate defense peptides responding to pathogenic intruders. They have attracted much attention on account of their broad-spectrum antibacterial property and less tendency to be resisted [13]. AMPs can be divided into α-helixes, β-folds, Cys-rich and other forms according to their structures, and can also be divided into natural AMPs and synthetic AMPs according to their sources [14]. A large number of natural AMPs have been detected and studied for their antibacterial efficacies, including oral-derived AMPs found in humans, such as β-defensin, which is expressed by oral epithelial cells as well as the salivary glands [15]; lactoferrin, which is found in most saliva neutrophils [16]; adrenomedullin, which is secreted by the oral epithelial cells [17]; calprotectin, which is expressed in the neutrophils, monocytes, macrophages, and epithelial cells [18]; and LL-37, which is a cathelicidin-derived AMP found in oral neutrophils as well as epithelial cells [19]. However, poor stability, a short half-life, a quick removal period and high cost are inevitable limitations of natural antimicrobial peptides. 

In our previous studies, we designed a new antimicrobial peptide, GH12 (Gly-Leu-Leu-Trp-His-Leu-Leu-His-His-Leu-Leu-His-NH_2_), which has a relatively low molecular weight of 1487.8 and a positive net charge of 4. The hydrophilic and hydrophobic amino acid residues are separately distributed on both sides of the helical wheel, leading to a typical amphiphilic α-helical structure [20]. It was found that GH12 can be adsorbed by anion sites on bacterial membranes through electrostatic interaction, and then GH12 disturbed the membrane through its α-helical structure and eventually caused depolarization or perforation of the membrane, thus playing an antibacterial role. According to our previous research, GH12 can effectively inhibit the growth and biofilm formation of *S. mutans* at the minimal inhibitory concentration (MIC) of 8 mg/L. In addition, we also found that the acid-producing ability of *S. mutans* significantly decreased after treatment with sub-MIC levels of GH12 (4 mg/L). RT-qPCR confirmed that the expression of genes related to the production of lactic acid (*ldh*), the synthesis of EPS (*gtfB*/*C*/*D*) and two-component signal transduction systems (*vicR*, *liaR*) decreased simultaneously compared with the untreated group [21]. Furthermore, we demonstrated that GH12 at the MIC not only reduced the proportion of *S. mutans* in a three-species bacteria model, but also inhibited the acidogenicity of the biofilm [22]. In another in vivo study, we topically applied 8 mg/L of GH12 to the teeth of rats with a camel hair brush for 5 min three times daily. The results proved that 8 mg/L of GH12 suppressed the onset and development of dental caries in rats [23]. Moreover, a stronger caries-controlling effect was observed when GH12 (8 mg/L) was combined with NaF (250 ppm) three times daily for 5 min each in a rodent model [24]. Therefore, we speculated that GH12 not only disturbed bacterial cell membranes but also directly affected the process of acid production in *S. mutans*. This activity might be attributed to its intracellular interaction with specific PEP-PTS pathways to arrest the transmembrane transportation of carbohydrates or its inhibition of certain enzymes related to carbohydrate metabolism. In addition, since GH12 performed better in the presence of fluoride, we hypothesized that as well as possible cellular activities to arrest the acid production of *S. mutans*, GH12 also provides fluoride with an extra ability to enter into bacterial cells through its perforating ability [24], and thus inhibited the acidogenicity of *S. mutans* more strongly. 

Thus, the purpose of this research was to verify the inhibitory efficacy of GH12 and NaF on the acid production of *S. mutans* biofilms and to reveal the underlying mechanisms. To mimic clinical application patterns, NaF, GH12 and their combination were applied to *S. mutans* biofilms for a short period. A lactic acid production assay was conducted, and the biofilm’s pH was monitored to detect the alterations in the bacteria’s acidogenicity. Transcriptome sequencing (RNA-seq) and bioinformatic analyses were conducted to explore the mechanisms, and RT-qPCR was used for validation. To the best of our knowledge, this is the first time that the synergy between and the mechanisms behind the antimicrobial peptide and NaF in suppressing the acidogenicity of *Streptococcus mutans* biofilms have been confirmed.

## 2. Materials and Methods

### 2.1. Peptide, Reagents and Bacteria

Peptides were synthesized and purified by GL Biochem (Shanghai, China). Following the Fmoc-solid phase peptide method, GH12 (Gly-Leu-Leu-Trp-His-Leu-Leu-His His-Leu-Leu-His-NH2) was synthesized. After being identified by liquid chromatography-tandem mass spectrometry, GH12 was further purified to 98%. GH12 was freeze-dried in powder form and stored at −20 °C. *Streptococcus mutans* UA159 samples were obtained from the State Key Laboratory of Oral Diseases at Sichuan University (Chengdu, China). *S. mutans* was grown in brain heart infusion (BHI) broth (Oxoid, UK) anaerobically (85% N_2_, 10% H_2_, 5% CO_2_) at 37 °C; 1% sucrose was added to the BHI broth for culturing the *S. mutans* biofilm. Unless otherwise stated, all chemicals were purchased from Solarbio (Beijing, China).

### 2.2. Biofilm Culture and Treatment

**Figure 1:**
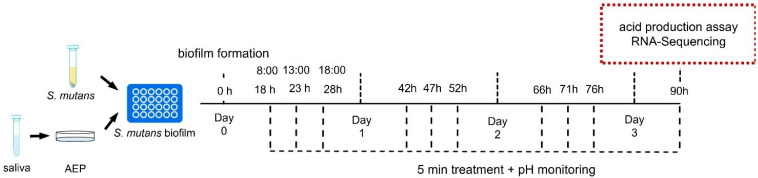
Design of the model of the short-term treatment of biofilm.

The *S. mutans* biofilms were cultured in six-well plates. Unstimulated whole saliva was acquired from five healthy caries-free volunteers (three male and two female) aged between 22 and 25 years old. All donors were instructed to abstain from food and drink for 30 min before sampling. The donors had no history of smoking and had been free from antibiotics for at least 3 months. The saliva was collected with the approval of the Institutional Review Board of the West China Hospital of Stomatology (WCHSIRB-D-2018-109) and sterilized with 0.45 μm filters. First, 200 μL of sterile saliva was added to each well and incubated at 37 °C for 2 h to form a mimetic acquired enamel pellicle (AEP). After the addition of 2 mL of BHIS containing 1 × 10^6^ CFU/mL of *S. mutans* to each well, the plates were incubated anaerobically at 37 °C for 18 h to form *S. mutans* biofilms. Double-distilled water (DDW) was used to treat the negative control group. The treatment groups included (1) 250 ppm of NaF, (2) 8 mg/L of GH12, and (3) 250 ppm of NaF and 8 mg/L of GH12 (the GF group). Three replicates were established for each group. All the reagents were diluted using DDW and were sterilized by filtration with 0.45 μm filters. A short-term treatment of 5 min was used in all the groups to mimic clinical treatment patterns (Figure 1). Briefly, 1 mL of the reagents was added to the corresponding wells for 5 min, and phosphate-buffered saline (PBS) was used for rinsing before and after the treatment.

### 2.3. Lactic Acid Production Assays

*S. mutans* biofilms were prepared and treated as mentioned above. After the treatment, the biofilm was gently washed twice with PBS to remove the planktonic bacteria and the residual medium. Next, 1 mL of buffered peptone water (BPW) with 0.2% sucrose was supplied to each well for 2 h of anaerobic incubation at 37 °C. The BPW supernatants were collected and then centrifuged to remove planktonic bacteria for an assessment of the lactic acid using a lactate assay kit (catalog number A019-2; Jiancheng, Nanjing, China). A spectrophotometer (Thermo Scientific, Waltham, MA, USA) was used to detect the absorbance at 570 nm. The concentration of lactic acid was calculated using the standard curve. The experiment was repeated three times.

### 2.4. Monitoring of the Biofilm’s pH

*S. mutans* biofilms were prepared in 24-well plates, as mentioned above. Short-term treatments were applied at certain time points. After rinsing with PBS, fresh medium was added to the wells. At each time point, supernatants of the medium were collected for testing the pH with a pH meter (Thermo Scientific, Waltham, MA, USA). The average value of three repeated tests from each well was considered to be the pH value of the sample. The experiment was repeated three times.

### 2.5. RNA Extraction, Library Preparation and RNA Sequencing

*S. mutans* biofilms were prepared in 24-well plates, as mentioned above. After 10 short-term treatments, the *S. mutans* biofilms were scraped gently from the plate. The samples were then centrifuged at 8000× *g* rpm, 4 °C, washed twice with PBS, and immediately frozen in liquid nitrogen for 4 h. The samples were then stored at −80 °C until later use. Three replicates were established for each group. 

For RNA extraction, TRIzol^®^ reagent (Invitrogen) was used to disrupt the cell membrane of the bacteria and inhibit the release of nuclease. After grinding under liquid nitrogen in fastrep tubes, the RNA of the samples was extracted with chloroform and separated by a high-salt solution (TaKara, Kusatsu, Japan) and isopropanol. After centrifugation, DEPC water was added to the samples according to the size of the precipitate at the bottom of the tubes. The genomic DNA was removed using DNase I (Takara). The extracted RNA samples were determined by an Agilent 2100 BioAnalyzer System, and the concentration was determined by a NanoDrop 2000. High-quality RNA samples (OD_260/280_ = 1.8–2.0, OD_260/230_ ≥ 1.0, 23S:16S ≥ 1.0, concentration ≥ 100 ng/μL, total amounts ≥ 2 μg) were used for subsequent construction of the RNA library.

The RNA library was constructed as described in the TruSeqTM RNA Sample Preparation Kit (Illumina, San Diego, CA, USA), including mRNA purification, mRNA fragmentation, cDNA synthesis and modification, library enrichment and sequencing. After amplification, the Picogreen dsDNA (TBS380) Kit was used to test the concentration of dsDNA and remove single-stranded DNA. The Agilent 2100 Bioanalyzer System was used to test the samples’ size and purity. The samples were mixed according to the proportions in the data and prepared for sequencing. 

The HiSeq X Ten kit v2.5 was prepared according to the manufacturer’s instructions, which contained the cluster reagents used for the cBot system, as well as the SBS reagents, labeling reagents and the double-end sequencing reagents used for the HiSeq X Ten kit. Bridge PCR amplification was performed on the cBot system to generate clusters. After inputting the operating parameters, loading SBS reagents with the fluidic system and installing the flow cells loaded with gene clusters, double-ended RNA sequencing was then performed using the Illumina HiSeq X Ten (2 × 150 bp) kit.

### 2.6. Bioinformatic Analysis

The sequencing results were converted to text signals as raw data in fastq format using CASAVA base recognition. The reference genome’s accession number is GCF_000007465.2. The amount of data prepared for quality control was at least 2 Gb, and the proportion of Q20 was higher than 90%. SeqPrep was used to remove splice sequences from the sequencing data, and Sickle (version 1.33) was used to filter the unqualified reads to obtain clean reads. The clean reads were mapped using the local alignment method in Bowtie2 (version 2.2.9) to obtain mapped data for the subsequent analysis. Basic functional annotation was performed on the reference genome, and the protein sequences were matched with six major databases, including the NR database, the Swiss-Prot database, the Pfam database, the EggNOG database, the GO database and the KEGG database, to obtain the corresponding functional and pathway annotations. The genes were annotated by all 6 databases, and the best-matched information of each database was taken as the annotation information. Gene expression levels were quantified using RSEM by calculating the transcripts per million reads (TPM). Then, Venn analysis, Pearson’s correlation analysis and principal component analysis (PCA) were performed according to the gene expression levels in different samples. DESeq2 software (version 1.24.0) was used to calculate the differentially expressed genes (DEGs) among the groups. The genes for which the adjusted *p* value and the BH multiple test correction result of the P-value were less than 0.05, and with |log_2_Fold Change| ≥ 1 were defined as the DGEs. The DEGs of interest from the four experimental groups were pooled as a gene set for further GO and KEGG enrichment analysis. 

### 2.7. Validation by Real-Time Quantitative Polymerase Chain Reaction (RT-qPCR)

The results of RNA-seq were verified by RT-qPCR. The *S. mutans* biofilms were cultured and treated as mentioned above. After extracting the total RNA from the samples, the PrimeScript^TM^ Reverse Transcription Kit (RR047A, Takara) containing the gDNA Eraser was used to synthesize the cDNA. The key DEGs were selected to conduct the RT-qPCR validation based on the GO and KEGG enrichment results, and the specific primers were designed using Primer3web version 4.1.0 (https://primer3.ut.ee/), as shown in the Supplementary Materials (Table S3). Each 25 μL PCR reaction mix contained 12.5 μL of SYBR^®^ Premix Ex Taq TM II (RR820A, Takara), 80 ng of cDNA, 1 μL of the forward primers and 1 μL of the reverse primers, both at 10 μM. The qPCR was performed on a CFX96 real-time reaction system (Bio-Rad, Hercules, CA, USA) with 30 cycles of thermal cycling (95 °C for 5 s, 60 °C for 30 s) as reported previously [25]. The transcription level of the 16S rRNA gene was taken as the internal reference, and the 2^−ΔΔCt^ method was used to calculate the fold change in gene expression. The experiment was repeated three times. 

### 2.8. Statistical Analysis

SPSS 20.0 (IBM, Armonk, NY, USA) was used for statistical analysis. The significance level α was set to 0.05. In the lactic acid production assay, the significance of the inter-group differences was assessed using one-way ANOVA, while the intra-group differences were assessed using Tukey’s HSD test. In the test of *S. mutans* biofilms’ pH, pairwise analysis of the pH values among groups at each time point was performed using Dunnett’s *t*-test. Sidak’s *t*-test was used for a statistical test of differential gene expression among groups, and the significance level α was set to 0.05.

Goatools was used for the GO enrichment analysis, and a *p*-adjust value of less than 0.05 (Fisher’s exact test) was considered to be significantly enriched for this GO function. For the KEGG pathway enrichment analysis, an R script was used to calculate the Rich factor, which is the ratio of the number of genes in the DEGs annotated to the pathway to the number of genes annotated to the pathway in all genes. When the *p*-adjust was less than 0.05, the KEGG pathway was considered to be significantly enriched. The false discovery rate (FDR) procedure of Benjamini and Hochberg [26] was used to correct for multiple hypothesis testing (FDR = 0.05). The total transcriptome data were uploaded to a public database (https://www.ncbi.nlm.nih.gov/sra/PRJNA913612, accessed on 18 December 2022).

## 3. Results

### 3.1. The Effects of the GF Combination on Arresting the Acidogenicity of S. mutans Biofilms

**Figure 2:**
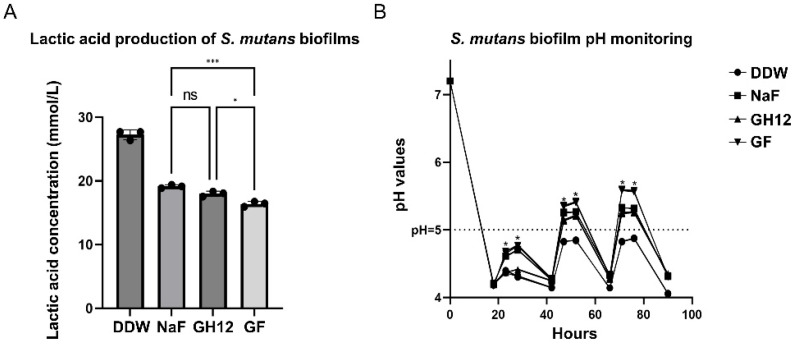
(**A**) The lactic acid concentration produced by *S. mutans* biofilms within 2 h after short-term treatment with each reagent. (**B**) The pH values of *S. mutans* biofilms at different time points after short-term treatment with each reagent. The results are the mean values from three repeated experiments. *: a significant difference was detected between the linked groups (*p* < 0.05). ***: a significant difference was detected between the linked groups (*p* < 0.001). ns—no significant difference.

The linear standard curves of lactic acid concentrations and OD_570_ values are provided in the Supplementary Materials (Figure S1). The lactic acid produced by *S. mutans* biofilms within 2 h is shown in Figure 2A. A significantly lower lactic acid concentration was detected in the GF group, which displayed lower lactic acid production than any of the individually applied treatments (*p* < 0.05). As illustrated in Figure 2B, all the experimental treatments expressed efficacy in delaying the decrease in the pH of *S. mutans* biofilms after the fresh medium was changed, and the GF group had better pH values that were higher than those of the other groups at all the tested time points (*p* < 0.05). Collectively, the variation in the pH of the GF group was consistent with its outcomes for lactic acid production, implying that the GF group has an outstanding ability to restrain the lactic acid production of *S. mutans* biofilms and thus delayed the pH drop of *S. mutans* biofilms.

### 3.2. RNA-seq and Analysis of Differentially Expressed Genes

The sequencing data were compared with the standard genome of *S. mutans* UA159 (GCF_000007465.2), as shown in Table S1 in the Supplementary Materials, resulting in a match rate of more than 97% with at least 94% of clean reads that matched the unique position of the reference gene set, thus reflecting the high reliability of the collected data. 

**Figure 3:**
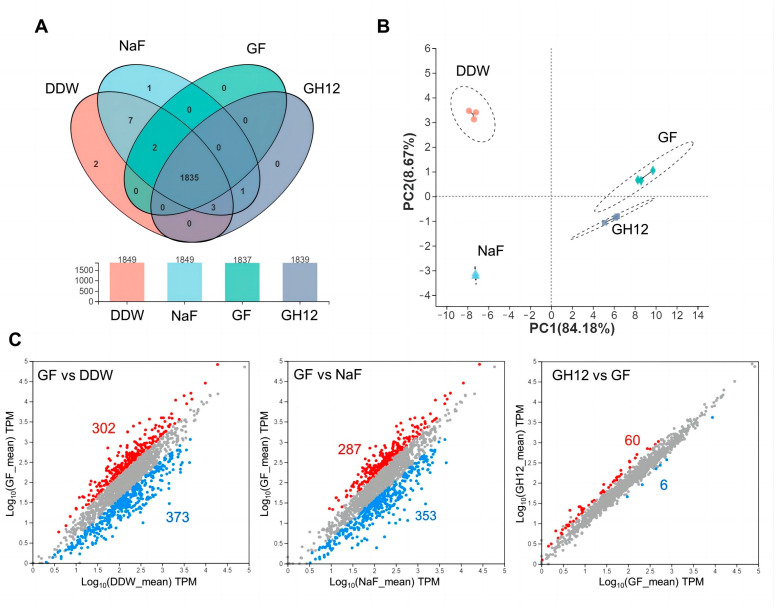
(**A**) Venn diagram of the number of genes in each group. (**B**) PCA of the samples. The distance between the groups refers to the similarity of the samples. (**C**) Scatterplot of the differentially expressed genes detected among groups. The red dots refer to significantly upregulated genes, and the blue dots refer to significantly downregulated genes.

As shown in Table S2 in the Supplementary Materials, 1859 genes in total were annotated in the public databases for all the groups. The Venn plot in Figure 3A shows that 1835 genes were co-expressed in all four groups, indicating that no wide gene mutation had occurred in any of the treatment groups. Moreover, the GF and GH12 groups displayed fewer gene transcription activities, hinting that the expression of certain genes might be inhibited in these two groups.

The PCA diagram in Figure 3B demonstrates that the DDW group and the NaF group displayed similar characteristics, while the GH12 group shared some similarities with the GF group. No mixed clustering was observed among the samples within the groups. The NaF group showed a negligible ability to induce transcriptional alterations in *S. mutans* biofilms alone. However, transcriptional alterations were widely observed when GH12 was applied, which further confirmed the intracellular functions of GH12. Moreover, the transcriptional difference between the GH12 and GF groups could be attributed to the additional application of NaF, which suppressed the expression of extra 60 genes. The DEGs of interest in the groups are illustrated as a scatterplot in Figure 3C. There were 675 DEGs between the GF group and the DDW group, of which 302 genes were significantly upregulated in the GF group, and 373 genes were downregulated in the GF group (*p*-adjust < 0.05). When we focused on the GF group and the NaF group, 640 DEGs were detected, of which 287 genes were upregulated in the GF group, and 353 genes were downregulated in the GF group (*p*-adjust < 0.05). Moreover, 66 DEGs were detected between the GF group and the GH12 group, of which 60 genes were downregulated in the GF group (*p*-adjust < 0.05), suggesting that additional application NaF led to the suppression of certain genes. Collectively, these findings were in agreement with previous research, showing that low concentrations of NaF had a minor effect on bacterial activities, and GH12 showed a stronger intracellular effect in comparison.

### 3.3. The Effects of the GF Combination on the Expression of Acid-Producing Genes of S. mutans Biofilms

**Figure 4:**
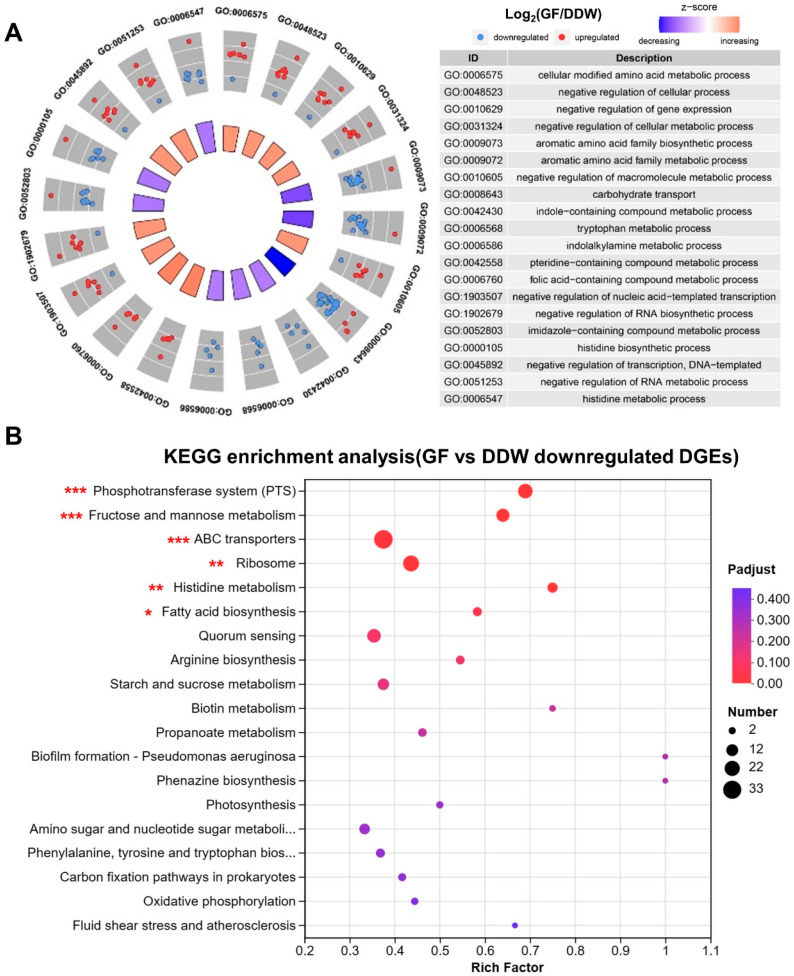
(**A**) GO enrichment circle diagram of DEGs between the GF group and the DDW group, showing the top 20 enriched GO functions. Downregulated and decreasing mean that the GF group showed downregulated gene expression or inhibited function compared with the DDW group. The table provides a description of the corresponding GO functions. (**B**) Bubble map of the KEGG enrichment pathways of the downregulated DEGs in the GF group (compared with the DDW group). The size of the dots indicates the number of genes enriched in this pathway, and the color of the dots refers to the *p*-adjust value, indicating the significance of the enrichment set. ***: FDR < 0.001; **: FDR < 0.01; *: FDR < 0.05.

DEGs between the GF group and the DDW group were used for the GO and KEGG enrichment analysis to explore the significantly enriched functions and pathways. As shown in Figure 4A, the top three downregulated GO pathways were GO:0008643 (carbohydrate transport), GO:0009072 (aromatic amino acid family metabolic process) and GO:0009073 (aromatic amino acid family biosynthetic process) (*p*-adjust < 0.05). The top three upregulated GO pathways were GO: 0042558 (pteridine-containing compound metabolic process), GO:0051253 (negative regulation of RNA metabolic process) and GO: 0006760 (folic acid-containing compound metabolic process) (*p*-adjust < 0.05). Collectively, the application of GF significantly arrests the transportation of carbohydrates and the amino acid metabolism of *S. mutans* biofilms. 

As shown in Figure 4B, six KEGG pathways were significantly downregulated by the GF group, of which the top three enriched pathways were the PEP-PTS pathways, fructose and mannose metabolism, and the ATP-binding-cassette transporters (ABC transporters) pathways (FDR < 0.05). These results were consistent with GO functional enrichment analysis, which showed that the application of GF inhibited the main carbohydrate transportation pathways of *S. mutans*. 

If we combine the outcomes of the GO/KEGG analyses, 18 genes related to carbohydrate transport were selected for subsequent RT-qPCR validation, as shown in Table 1. The names and description of the functions of the genes selected for RT-qPCR are listed in the first and second columns. The ratios of the TPM reads of GF/DDW in the third column demonstrate that the GF group showed lower gene expression levels when the ratios were below 1. Moreover, the outcomes of RT-qPCR (2^−ΔΔCT^) in the fourth column indicated a lower gene expression level for the GF group when the value was less than 1. Thus, the outcomes of RT-qPCR validated the results of the RNA-seq analysis (*p* < 0.05). They collectively confirmed that the GF group significantly downregulated the genes related to the transmembrane transport of carbohydrates.

### 3.4. The Additional Effects of the GF Combination on S. mutans Biofilms Compared with Applying GH12 or NaF Alone

**Figure 5:**
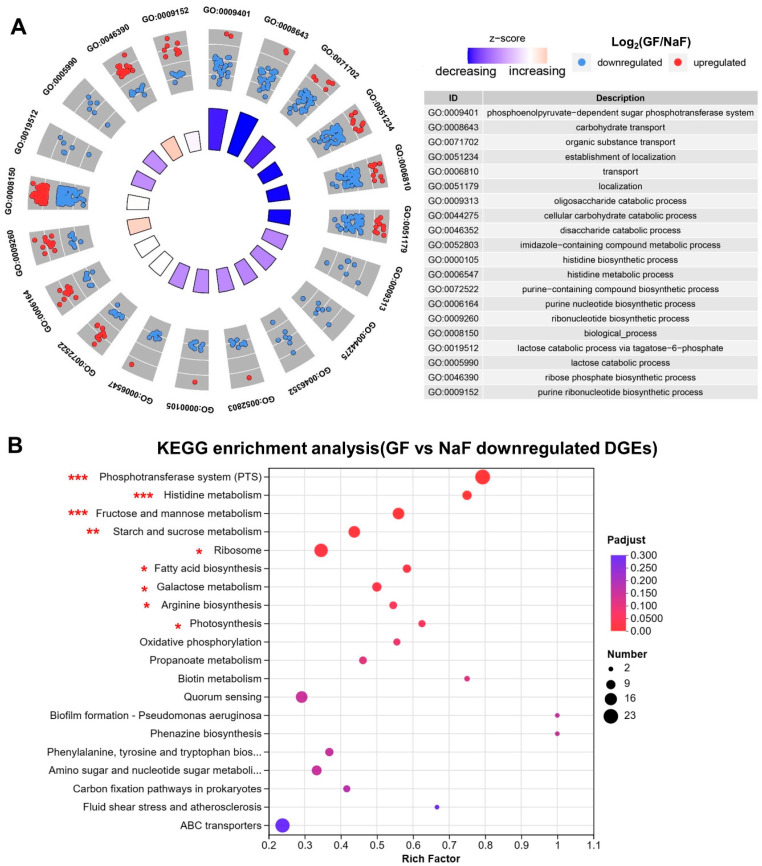
(**A**) GO enrichment circle diagram of the differentially expressed genes between the GF group and the NaF group, showing the top 20 enriched GO functions. Downregulated and decreasing mean that the GF group showed downregulated gene expression or inhibited function compared with the NaF group. Upregulated and increasing mean that the GF group showed upregulated gene expression or increased function compared with the NaF group. The table provides a description of the corresponding GO functions. (**B**) Bubble map of the KEGG enrichment pathways of the downregulated DEGs in the GF group (compared with the NaF group). The size of the dots indicates the number of genes enriched in this pathway, and the color of the dots refers to the *p*-adjust value, indicating the significance of the enrichment set. ***: FDR < 0.001; **: FDR < 0.01; *: FDR < 0.05.

The 640 DEGs between the GF group and NaF group were pooled as a gene set for further GO and KEGG enrichment analyses (Figure 5). According to the GO enrichment analysis (Figure 5A), the GF group showed less activity in certain functions, such as GO:0009401 (PEP-PTS), GO:008643 (carbohydrate transport), GO: 0071702 (organic substance transport), GO:0051243 (establishment of localization), GO:0006810 (transport) (*p*-adjust < 0.05). These downregulated functions were consistent with the outcomes of the KEGG analysis (Figure 5B). Thus, compared with the application of NaF alone, the GF group exhibited enhanced inhibition of PEP-PTS, transportation of carbohydrates and other catabolic processes. 

**Figure 6:**
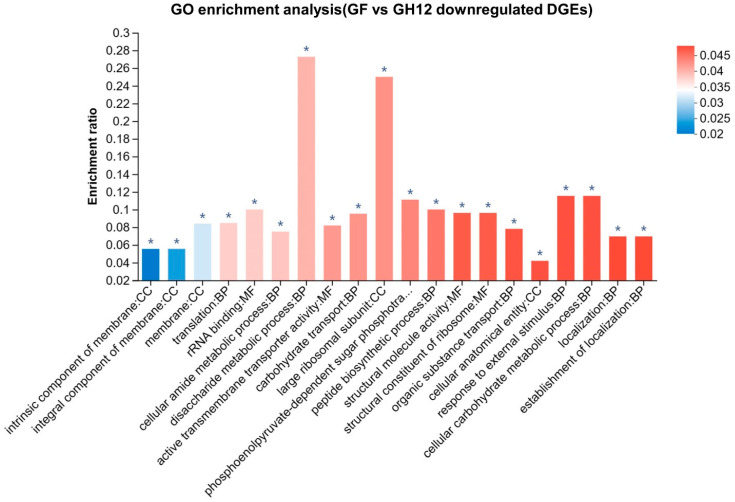
GO enrichment bar plot of 60 downregulated DGEs in the GF group compared with the GH12 group; *: *p*-adjust < 0.05.

To explore the contribution of NaF in the GF combination, 60 downregulated DEGs in the GF group (compared with the GH12 group) were established as a gene set for GO enrichment analysis (Figure 6). Compared with the GH12 group, the GF group showed a notable suppression of membrane integration, the ribosome, response to external stimulus, disaccharide metabolic processes, and cellular carbohydrate metabolic processes (*p*-adjust < 0.05). These results showed that the GF group further disturbed the intracellular environment of *S. mutans* with the help of NaF, which led to a further influence on the processes of sugar metabolism. 

## 4. Discussion

The purpose of our research was to verify the efficacy of the GF combination on the acidogenicity of *S. mutans* biofilms and to reveal the underlying mechanisms. The lactic acid production assay and the pH test together led to the conclusion that GF showed better efficacy in arresting the acidogenicity of *S. mutans* biofilms than the DDW group and the individual treatment groups. Moreover, the RNA-seq and bioinformatics analyses demonstrated that GF suppressed the expression of specific PTS-related genes and metabolism-related genes of *S. mutans* biofilms thus inhibiting the transportation of carbohydrates and metabolism.

Known as the principal bacteria responsible for the development of caries, *S. mutans* takes advantage of its outstanding acidogenic and aciduric properties. Lactic acid has been reported as the major final acidic metabolite of the carbohydrate metabolism of *S. mutans* and is believed to be responsible for the notable drop in the pH of the micro-environment of dental plaque [27]. According to Figure 2, the GF group performed better in arresting the production of lactic acid compared with the other groups. Moreover, as the predominant end product of carbohydrate fermentation, lactic acid indirectly reflects the metabolic state of bacteria [28]. Thus, the decrease in the production of lactic acid by *S. mutans* biofilms indicated that the application of these agents put extra stress on *S. mutans* biofilms and led to an impaired metabolic status. In support of this, the GF group displayed a notable ability to inhibit the decrease in the pH of *S. mutans* biofilms. These facts indicated that the GF combination was able to suppress the overall acidogenicity of *S. mutans* biofilms.

Prokaryotic RNA-seq utilizes high-throughput sequencing technology to enable an accurate annotation of the transcriptome’s features and has been widely used to determine the differential expression of different strains and/or conditional transcripts [29]. As shown in Figure 3, among all 1859 annotated coding genes, there were 1835 co-expressed genes in the four groups, indicating that the treatments caused no wide gene mutations. The PCA revealed that the GH12 group and the GF group showed similar but well-demarcated characteristics, which were distinct from those of the DDW and NaF groups, indicating that NaF alone induced minor transcriptional alterations of *S. mutans* biofilms. This correlates with the theory that although fluoride has some intracellular efficacy, it faces a barrier against entry into the cell. In addition, the difference between the GF and GH12 groups might be attributed to the addition of NaF. 

The PTS is a series of carbohydrate-specific transporters that dominate the active transport of certain carbohydrates in Gram-positive bacteria [30]. As saccharolytic organisms, caries-related pathogens, especially *S. mutans*, is highly dependent on PTS not only to fulfill the energy demands for metabolism and reproduction but also to create an acid microenvironment and thus gain a growth advantage through further fermentation of internalized carbohydrates [31]. The general suppression of carbohydrate transport according to the GO/KEGG analysis (Figure 4) and validation of the related genes through qPCR (Table 1) indicated that the overall carbohydrate transport and metabolism of *S. mutans* biofilms were inhibited by the GF combination. 

Besides, the ABC transporter was also downregulated by the GF group (Figure 4). It is widely recognized that ABC transporters are responsible for the uptake of disaccharides and oligosaccharides [32]. The knockdown of two ABC transporter proteins, SMU0836 and SMU0837, led to the increased sensitivity of *S. mutans* to antibiotics [33]. Moreover, the inhibition of CslAB, an ABC transporter, impaired the ComDE density-sensing system in *S. mutans* [34]. Thus, inhibiting the ABC transporter’s function can lead to the impaired efflux of bacteria in response to drugs, toxins, and other stressors. On the basis of the points above, it was speculated that GF not only inhibits the metabolic processes of *S. mutans* biofilms but also limits the efflux of GH12 and NaF, so that the GF group had a greater effect on the functional activity of *S. mutans* than GH12/NaF applied alone.

Under the GF treatment, an increase in the synthesis of nucleic acids of *S. mutans* biofilms was observed, while RNA transcription and carbohydrate transportation were suppressed, hinting that *S. mutans* biofilms attempted to carry out energy-consuming processes of synthesis even when the energy metabolism was insufficient. The abnormal employing of energy reminded us that the bacteria were experiencing severe environmental stress and tried to initiate pathways such as DNA repair or resistance to oxidative stress [35]. However, such efforts seemed vain since the subsequent transcription and translation were inhibited.

The PTS pathways were originally reported to be one of the intracellular targets of fluoride [36]. The GO and KEGG analysis of the DEGs between the GF group and the NaF group (Figure 5) revealed the enhanced suppression of the PTS pathways and sugar metabolism by the GF group. It was deduced that GH12 disrupted the cell membrane’s integrity, which allowed more fluoride to act on the subunit of PTS and enter the bacterial cell to affect the metabolism. As shown in Figure 6, compared with the GH12 group, the GF group inhibited the membrane’s integration, ribosomes, the response to external stimuli, metabolic processes of disaccharides and cellular carbohydrates. These results showed that the GF group further disturbed the intracellular environment of *S. mutans* with the help of NaF, which led to a further influence on the processes of sugar metabolism.

In summary, combining GH12 and fluoride could arrest the acid production of *S. mutans* biofilms, mainly by suppressing the PTS-related transportation of carbohydrates and thus inhibiting the overall metabolism. However, as caries is a multifactorial disease, arresting the acidogenicity of *S. mutans* may make convincing but also limited contributions to caries control. Other measures, such as maintaining oral hygiene or adopting healthy dietary habits, also play vital roles in preventing caries. Moreover, most dental caries were found to have already formed in clinical circumstances. Antibacterial measures that can prevent the onset of caries or halt the development of caries are clearly not enough to treat caries. Although fluoride has proven to be helpful in rebalancing mineral homeostasis, other measures that enhance the remineralization of impaired enamel are also ideal strategies for treating caries, especially those that provide biomimetic mineralization of caries lesions such as Zinc Hydroxyapatite [37,38], Ca/P-PILP [39] and TiO_2_-HAP nanoparticles [40]. Thus, future research should focus on building a strategy to prevent and treat caries at the same time, probably by adopting antibacterial agents and biomimetic mineralizing agents as sequenced treatments.

## 5. Conclusions

Collectively, our work demonstrated that the combination of the antimicrobial peptide GH12 and fluoride showed promising efficacy in arresting the acidogenicity of *S. mutans* biofilms, mainly by suppressing PTS-related genes and inhibiting bacterial metabolism. The combination exhibited better efficacies than any other individually applied treatments. However, antibacterial strategies face limitations in the prevention and treatment of caries. As well as maintaining oral hygiene and healthy dietary habits, biomimetic measures promoting mineralization as a sequenced treatment are also a promising direction for future surveys.

## Figures and Tables

**Table 1:** RT-qPCR validation of the genes of interest related to the transmembrane transport system (GF vs. DDW) (*: *p* < 0.05).

Gene Name	Gene Description	Fold Change (GF/DDW)	2^−ΔΔCt^ (mean ± SD)
SMURS07250	BglG family transcription antiterminator	0.06	0.135 ± 0.096 *
SMURS00600	Fructose PTS transporter subunit IIA	0.104	0.112 ± 0.005 *
SMURS00595	Fructose-specific PTS transporter subunit EIIC	0.114	0.106 ± 0.005 *
celB	PTS cellobiose transporter subunit IIC	0.389	0.368 ± 0.014 *
SMURS01555	PTSglucitol/sorbitoltransportersubunit IIA	0.361	0.308 ± 0.035 *
SMURS01550	PTSglucitol/sorbitoltransportersubunit IIB	0.291	0.287 ± 0.003 *
SMURS01545	PTS glucitol/sorbitol transporter subunit IIC	0.285	0.370 ± 0.057 *
SMURS08595	PTS mannose/fructose/sorbose transporter subunit IIC	0.148	0.192 ± 0.030 *
SMURS01535	PTS sugar transporter subunit IIA	0.398	0.184 ± 0.143 *
SMURS00525	PTS sugar transporter subunit IIB	0.395	0.176 ± 0.152 *
SMURS08590	PTS sugar transporter subunit IIB	0.095	0.134 ± 0.036 *
SMURS08905	PTS sugar transporter subunit IIB	0.068	0.176 ± 0.137 *
SMURS08900	PTS sugar transporter subunit IIC	0.093	0.220 ± 0.142 *
SMURS08600	PTS system mannose/fructose/sorbose family transporter subunit IID	0.164	0.223 ± 0.092 *
SMURS08895	PTS system mannose/fructose/sorbose family transporter subunit IID	0.112	0.146 ± 0.095 *
treP	PTS system trehalose-specific EIIBC component	0.073	0.187 ± 0.126 *
SMURS09355	PTS transporter subunit IIBC	0.357	0.246 ± 0.152 *
SMURS08435	Sucrose-specific PTS transporter subunit IIBC	0.207	0.200 ± 0.118 *

## Data Availability

The transcriptomic data presented in this study are openly available from [https://www.ncbi.nlm.nih.gov/sra/PRJNA913612, accessed on 18 December 2022].

## Supplementary Materials

The following supporting information can be downloaded at: https://www.mdpi.com/article/10.3390/microorganisms11071796/s1, Figure S1: Standard curve of lactic acid concentration (calculated by corresponding OD570 values); Table S1: Matching rate of sequencing data between samples and standard *S. mutans* genome (UA159, GCF_000007465.2); Table S2: Gene annotation results in public databases; Table S3: Primers used in RT-qPCR validation.
