# Supplementary material for: A Transcriptional Analysis Showing the Effects of GH12 Combined with Fluoride for Suppressing the Acidogenicity of Streptococcus mutans Biofilms
Source: Microorganisms. 2023 Jul 13;11(7):1796. doi: 10.3390/microorganisms11071796 (PMC10386188; doi:10.3390/microorganisms11071796)
Supplement: Supplementary file 1 [file microorganisms-11-01796-s001.zip › microorganisms-2454908-supplementary materials.pdf]

Supplementary materials

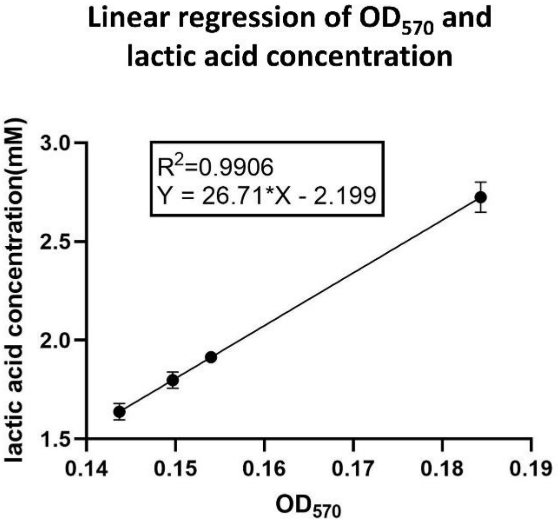

Figure S1. Standard curve of lactic acid concentration (calculated by corresponding OD570 values).

Table S1. Matching rate of sequencing data between samples and standard S. mutans genome (UA159, GCF_000007465.2).

Group	Sample	Total Reads	Matching Reads	Match Ratio（%）	Unique Match Reads	Unique Matching Ratio
DDW	A1	25231250	24956438	98.91	24315232	96.37
	A2	22978224	22753409	99.02	22164657	96.46
	A3	25068608	24800985	98.93	24119911	96.22
NaF	B1	25568554	25121039	98.25	24192081	94.62
	B2	25512706	25237549	98.92	24241382	95.02
	B3	22706512	22486435	99.03	21600828	95.13
GF	C1	25195716	24819503	98.51	24429449	96.96
	C2	24948830	24659537	98.84	24253282	97.21
	C3	25114286	24586272	97.9	24043036	95.73
GH12	D1	22308348	22045956	98.82	21600551	96.83
	D2	22240238	21929503	98.6	21492458	96.64
	D3	22760026	22516634	98.93	22062363	96.93

Table S2. Gene annotation results in public databases.

	Encoding genes	NR database	Swiss-Prot database	Pfam database
Annotated genes	1859	1858	1410	1706
		COG database	GO database	KEGG database
genes		1634	1537	1122

Table S3. Primers used in RT-qPCR validation.

Gene name	Primer Forward 5'-3'	Primer Reverse 5'-3'
16S rRNA	AGCGTTGTCCGGATTTATTG	CTACGCATTTCACCGCTACA
SMU_RS00525	TGACAGTTGTTGAGGCTAGAGT	ACTCATGCTTTTCCCAGAGG
SMU_RS00595	TGTGCATTTGACATGGGTGG	AAAAGCAGTGGTCGCAACAA
SMU_RS00600	TGCCATTCCTCATGCCAAAG	ATGTGTTGTCCCTGCTTCCT
SMU_RS01545	TGCCGGAGTATTACAAGCCA	ATCAGTCACCCAGCCAGAAA
SMU_RS01550	ATTGTTGACTGTGGTGGCAC	CTGGTATCATAGGTGCCGGT
SMU_RS01555	AAAAGTGCTGGCTGTTGGAC	ATAACAAGATGCCCGCCCTT
SMU_RS01535	TGCCGGAGTATTACAAGCCA	GCACCCAAGCTAACCAAAGT
celB	ATCCTCTAAACGGGGCTCTG	ATCCCTGTTGTGCCAAATCG
SMU_RS07250	CGTAAAGAGCTCGTCATGGC	GGACTGAAAATCGGCATGCT
SMU_RS08435	TCAGACAATGGTGCGGAGAT	TCCTGAAGAAGCGAGGGTTT
SMU_RS08590	CAATTGAAGGCGGTGTTCCA	CGTTTGGCACTTTACGGACA
SMU_RS08595	CTCATCATCGCCTTCCTTGC	GCCCAACCAAGAGCAAGAAA
SMU_RS08600	ACAAAGAAGATCGTGCTGCC	TTGGGCGAAGAACAAACCAG
SMU_RS08895	CTTGCGTGCAGGTCTTATGG	GGCTTGTCCTTGTGTTCCAA
SMU_RS08900	GGTCTTGGCGGTACAACAAT	CGCACATCAGCAGAAACAGT
SMU_RS08905	GCGAAAGAATACCCATGCGA	ACTTCACCCGGAACAAAACC
treP	TCTGAAGCGGTTGATCCTGT	TTCCAGATTGACCGTGTCCA
SMU_RS09355	ACAACATACCGCACCGTTTC	GCACCCAAGCTAACCAAAGT
